# Staying fit and the obese aging heart condition

**DOI:** 10.18632/aging.203070

**Published:** 2021-05-13

**Authors:** Chanisa Thonusin, Siriporn C. Chattipakorn, Nipon Chattipakorn

**Affiliations:** 1 Cardiac Electrophysiology Research and Training Center, Faculty of Medicine, Chiang Mai University, Chiang Mai, Thailand; 2 Cardiac Electrophysiology Unit, Department of Physiology, Faculty of Medicine, Chiang Mai University, Chiang Mai, Thailand; 3 Center of Excellence in Cardiac Electrophysiology Research, Chiang Mai University, Chiang Mai, Thailand; 4 Department of Oral Biology and Diagnostic Sciences, Faculty of Dentistry, Chiang Mai University, Chiang Mai, Thailand

**Keywords:** aging, obesity, cardiovascular disease, mitochondria, cardiorespiratory fitness

Cardiovascular disease has been and will continue to be a leading cause of morbidity and mortality, contributing to substantial social and economic burdens worldwide. Aging and obesity have been identified as two common risk factors of cardiovascular disease [1]. Although both conditions result in the development of cardiovascular disease independently [1], our previous study demonstrated that the combination of aging and obesity causes cardiac function to deteriorate even further [2]. In an experimental D-galactose-induced aging model, aging plus obesity led to greater severity of cardiac dysfunction when compared to either aging or obesity alone [2]. This dysfunction was mainly mediated by aberrant cardiac mitochondrial dysfunction, together with worsening autophagic impairment, increased oxidative stress, and increased apoptosis [2].

Unfortunately, the elderly obese population has risen globally, most probably due to the failure of weight control in young and middle-aged obese individuals, as well as the increased risk of obesity that comes with aging. These may contribute to increased incidence, prevalence, and severity of cardiovascular disease among the elderly. Therefore, attenuation of the aged-obese condition would be extremely beneficial in mitigating the burden of cardiovascular disease. Extensive research has been done to explore cardioprotective benefits of pharmacological interventions. It has been shown that anti-diabetic drugs such as metformin, and DPP4 and SGLT2 inhibitors are effective in alleviating cardiac dysfunction partly due to restoration of the obese associated impaired mitochondrial function, resulting in a decreased risk of obesity-induced cardiovascular disease [3]. Lifestyle modification of caloric restriction and exercise training are conventional strategies which have proved successful for weight loss and increased longevity [1]. These two interventions are considered key methods in amelioration of the combined aged-obese condition, potentially attenuating both obesity- and aging-induced cardiac dysfunction and cardiovascular disease [1]. Mechanistically, both caloric restriction and exercise improve cardiac mitochondrial function, leading to improved left ventricular function in the obese condition [4]. In reality, however, it is difficult to maintain caloric restriction and exercise training for one’s entire life due to the physiological adaptation to starvation and decreased exercise performance with age. All of these factors increasingly lead to a phenomenon known as “weight regain” after successful weight loss. Consequently, an alternative strategy that simultaneously exerts a protective effect against obesity, cardiovascular disease, and mortality becomes a research topic of interest in this field and “cardiorespiratory fitness” has become an attractive strategy among investigators [5].

Cardiorespiratory fitness is defined as the ability of the circulatory, respiratory, and muscular systems to supply oxygen during sustained physical activity. Although exercise training can increase cardiorespiratory fitness, this effect is not permanent since ∼72% of cardiorespiratory fitness is determined by genetics [6]. Previous longitudinal studies in humans have shown that intrinsically high levels of cardiorespiratory fitness result in decreased risk of weight gain, obesity, hypertension, myocardial infarction, heart failure, atrial fibrillation, cardiovascular mortality, and mortality from all causes [5]. Indeed, it has been demonstrated that cardiorespiratory fitness can predict the incidence of obesity, cardiovascular disease, and mortality [5]. Interestingly, these predictive effects were independent of age, body mass index, smoking, alcohol drinking, and the amount of physical activity [5].

The molecular mechanisms underlying the predictive effects of cardiorespiratory fitness on the development of obesity and cardiovascular disease have been widely studied in rats selectively bred for high and low intrinsic running capacity. The results from these studies suggest that high cardiorespiratory fitness decreases risk of obesity via alleviation of insulin resistance and disruptive nutrient oxidation [7]. The impairment of mitochondrial respiration, increased oxidative stress, and adverse cardiac remodeling are factors mediating the aggravating effect of the combined aged-obese condition on cardiac dysfunction [2], however high cardiorespiratory fitness has been shown to effectively mitigate those adverse effects, resulting in improved cardiovascular outcome in that condition [8]. Taken together, high cardiorespiratory fitness may counteract the harmful effects of the combined condition on the heart, and consequently contribute to the decreased risk of cardiovascular disease and mortality in the elderly obese population. Therefore, any novel paradigms that can directly target the enhancement of intrinsic cardiorespiratory fitness should be explored.

Currently, the relationships between the protective effect of high cardiorespiratory fitness and the detrimental effect of the combined aged-obese condition on the development of cardiovascular disease are not completely understood and further investigations are needed prior to the establishment of novel targeted therapies. Mitochondria play an important role in the pathophysiology of cardiovascular disease, therefore cardiac mitochondrial alterations which mediate the predictive effect of cardiorespiratory fitness on the incidence of cardiovascular disease should be holistically evaluated. These mechanisms include mitochondrial metabolism, mitochondrial oxidative phosphorylation and respiration, mitochondrial dynamics, mitochondrial oxidative stress, mitochondrial membrane potential change, mitochondrial swelling, and mitophagy. Additionally, a future study directly comparing the effect of cardiorespiratory fitness on a variety of cardiac parameters under various conditions including physiological condition, obesity, and aging should be performed. The results from such studies will reveal the strength of predictive effects of cardiorespiratory fitness on the development of cardiovascular disease in each condition. The use of cardiorespiratory fitness as a predictor of the incidence of cardiovascular disease should also be determined in subjects who receive either caloric restriction or exercise training. This will help identify the interactions between intrinsic cardiorespiratory fitness, caloric restriction, and exercise training. These results may be valuable on transference to future clinical practice to increase intrinsic cardiorespiratory fitness, further reducing the burden of cardiovascular disease, especially in the era when this is an issue across the world.
